# Effect of gaseous ozone treatment on cells and biofilm of dairy *Bacillus* spp. isolates

**DOI:** 10.3389/fmicb.2025.1538456

**Published:** 2025-03-17

**Authors:** Angela Maria Catania, Alessandra Dalmasso, Patrizia Morra, Emanuele Costa, Maria Teresa Bottero, Pierluigi Aldo Di Ciccio

**Affiliations:** ^1^Department of Veterinary Sciences, University of Turin, Turin, Italy; ^2^Department of Earth Sciences, University of Turin, Turin, Italy

**Keywords:** gaseous ozone, antimicrobial biofilm, *Bacillus cereus*, *Bacillus subtilis*, food contact surfaces

## Abstract

*Bacillus* spp. can produce biofilms and cause recurrent contamination in the food industry. The common clean-in-place (CIP) method is usually employed in sanitizing processing equipment. However, CIP is not always effective in removing biofilms. Ozone represents a promising “green” alternative to control biofilms. In this study, the effect of gaseous ozone (50 ppm) was evaluated *in vitro* against planktonic and sessile *B. cereus* and *B. subtilis* isolates collected from the dairy sector. Planktonic cells were enumerated by plate counts after 10 min, 1 h, and 6 h of ozone treatment. After a short-term (10 min) exposure, a slight reduction in microbial loads (0.66–2.27 ± 0.15 Log_10_ CFU/mL) was observed for *B. cereus* strains, whereas a more pronounced reduction (2.90–3.81 ± 0.12 Log_10_ CFU/mL) was noted in *B. subtilis* isolates. The microbial load further decreased after 1 h-treatments, around 1.5–3.46 ± 0.11 Log_10_ CFU/mL for *B. cereus* strains, and 4.0–5.6 ± 0.11 Log_10_ CFU/mL for *B. subtilis* isolates, until complete inactivation of bacterial cells after 6 h of exposure. Moreover, the effect of gaseous ozone treatment (50 ppm, 6 h) was evaluated for its ability to inhibit and eradicate biofilms formed on two common food-contact materials (polystyrene and stainless steel). Sessile *B. subtilis* cells were the more sensitive to the action of ozone, while a weak effect was highlighted on *B. cereus* isolates on both surface types. These results were further confirmed by scanning microscopy analysis. The number of cells in the biofilm state was also assessed, showing a not-complete correlation with a decrease in Biofilm Production Indices (BPIs). These findings highlighted the effectiveness of the sanitizing protocol using gaseous ozone in contrasting Bacillus free-living cells, but a not completely counteraction in biofilm formation (inhibition) or eradication of pre-formed biofilm. Thus, the application of ozone could be thought of not alone, but in combination with common sanitization practices to improve their effectiveness.

## 1 Introduction

The colonization of food plants by *Bacillus* spp. is of great concern in dairy environments since they could be a source of recurrent contamination of dairy products (Gopal et al., 2015). Among *Bacillus* spp., *B. cereus* is one of the most relevant foodborne pathogens, able to produce toxins that cause diarrhea and emetic illness (Jessberger et al., 2020). *B. subtilis* is an important spoilage bacterium in dairy processing plants since it can produce protease and lipase enzymes which are responsible for organoleptic changes in dairy products (Chen et al., 2003; Kumari and Sarkar, 2014). *Bacillus* spp. are spore-former bacteria, and spores exhibit a solid attachment to dairy processing equipment (Watterson et al., 2014). Both bacterial species are ubiquitous and known for producing biofilm on various surfaces, such as plastic, stainless steel, and aluminum, commonly used in dairy plants (Hayrapetyan et al., 2015; Catania et al., 2023). Biofilm protects bacterial cells against external environmental stresses, and it is challenging to remove (Roy et al., 2018; Sharma et al., 2019; Muhammad et al., 2020).

A high level of hygiene is essential in the food industry to prevent spoilage and contamination by foodborne bacteria. If working surfaces and equipment are not properly sanitized, residues of organic materials may persist causing favorable conditions for the development of microbial biofilm (Sharma et al., 2023).

The most common system to sanitize dairy equipment and machinery is the automated cleaning/disinfection procedure called “cleaning in place program” (CIP). Based on alkaline/acid cleaning with hot water disinfection, the CIP system is used for routine procedures (Srey et al., 2013). Considering the significant environmental impact of the CIP system, due to large water consumption and huge amount of wastewater produced, sustainable technologies should be encouraged. Regarding this, there has been growing interest in using ozone gas treatment to prevent and/or eradicate established bacterial biofilm (Epelle et al., 2023).

Ozone is a triatomic allotropic oxygen modification (Piletić et al., 2022.). Contrary to conventional disinfectants, it does not leave residues, since it rapidly decomposes to oxygen (Canut and Pascual, 2008). Ozone is characterized by high antimicrobial activity against a wide range of Gram-positive and Gram-negative bacteria (Guzel-Seydim et al., 2004). In 2001, the Food and Drug Administration (FDA) recognized the employment of ozone in gaseous and aqueous phases as antimicrobial agents in the food sector (U.S. Food and Drug Administration, 2024). In particular, the ozone in the gaseous phase can easily reach areas of the food equipment called “dead zones” such as pits, cracks, and recesses where biofilm may accumulate and that are difficult to sanitize by using cleaners and/or disinfectants in the aqueous phase (Moat et al., 2009). However, as far as the use of ozone gas in food processing environments is concerned, concentration and exposure time to ozone must be considered, since exposures around 0.1 ppm can have negative health effects on humans such as irritation to the eyes and respiratory system, headaches, dry throat, etc. (Piletić et al., 2022; Botondi et al., 2023). Therefore, ozone gas treatments should be carried out in the absence of food handlers and scheduled during the plant’s weekly shutdown periods.

The current study aimed to evaluate the effect of gaseous ozone against bacterial cells and biofilm produced by dairy *B. cereus* and *B. subtilis* isolates on food contact surfaces commonly used in dairy processing plants.

## 2 Materials and methods

### 2.1 Bacterial strains

Eight *Bacillus* spp. isolates (four *B. cereus* and four *B. subtilis* strains), previously isolated in an Italian dairy plant located in Piedmont and characterized by MALDI-TOF and rRNA 16 s sequencing (Catania et al., 2021), were used for experimental trials (Table 1). Reference strains *B. cereus* ATCC 14579 and *B. subtilis* NCIB 3610, were also included in this study.

**Table 1:** Dairy *Bacillus* isolates included in the study.

Strain ID	Species	Collection period	Sequence type (ST)
BC_2	*B. cereus*	Summer	ST-32
BC_14		Summer	ST-371
BC_36		Autumn	ST-371
BC_44		Autumn	ST-371
BS_8	*B. subtilis*	Summer	ST-249
BS_23		Summer	
BS_42		Autumn	
BS_54		Autumn	

Species identification was performed by MALDI-TOF and confirmed by rRNA 16 s sequencing analysis. They were collected in an Italian dairy plant during the summer and autumn seasons 2020. The ST was determined by MLST allelic profiles.

### 2.2 Ozone treatment chamber

The system employed to assess the effect of gaseous ozone consisted of a sealed plexiglass chamber (Biofresh Group Ltd., Northumberland, UK) connected to an ozone generator (Model-LF5; Biofresh Group Ltd., Northumberland, UK). The injection and concentration of gaseous ozone in the chamber were regulated and monitored by an ozone analyzer (UV-100, EcoSensor, Santa Fe, USA). A fan placed in the chamber ensured widespread gas distribution during the treatments. Since a synergistic effect between ozone and high relative humidity (%RH) (≥ 50%) has been documented (Epelle et al., 2023), bowls with warm water were placed on the chamber’s bottom to maintain RH high (≥ 90%). A portable data logger (Testo 174 H, Testo AG, Lenzkirchen, Germany), was used to monitor room temperature and humidity. Experiments were performed in triplicate at room temperature.

### 2.3 Gaseous ozone treatment against *Bacillus cereus* and *Bacillus subtilis* cells

**Figure 1:**
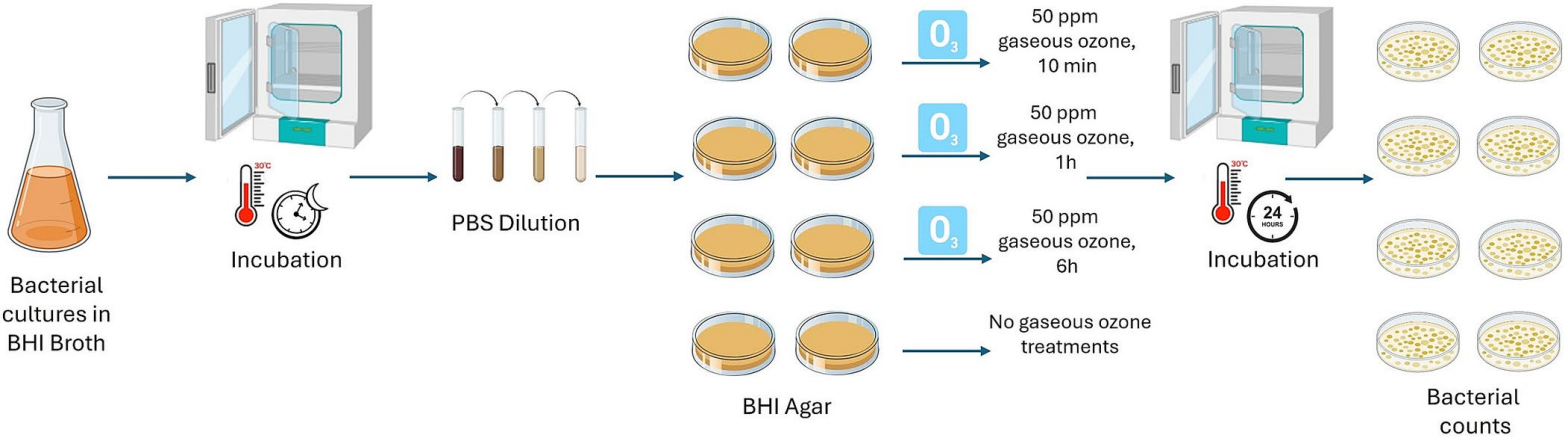
Schematic representation of 50 ppm gaseous ozone treatments on cells of dairy-related *B. cereus* and *B. subtilis* strains, including reference strains, at different exposure times. This image was created with BioRender (https://biorender.com/).

Firstly, the action of ozone gas was evaluated, at different exposure times, on *B. cereus* and *B. subtilis* cells without a protective layer of a polymer, typically found when bacterial cells are organized in a biofilm state. The reference strains *B. cereus* ATCC 14579 and *B. subtilis* NCIB 3610 were included in the experiment. Briefly, *B. cereus* and *B. subtilis* isolates were pre-cultured in BHI broth (Oxoid, Basingstoke, UK) at 30°C overnight. Cultures were diluted (1:10) in sterile PBS and appropriate dilutions were plated on BHI Agar (BHA; Oxoid, Basingstoke, UK) in duplicate. Then, inoculated plates were treated with gaseous ozone at 50 ppm for 10 min, 1 h, and 6 h. Inoculated BHA plates not submitted to ozonization were used as controls. To enumerate the cells, treated and control plates were incubated at 30°C for 24 h. After ozone exposure, bacterial counts were performed to evaluate the logarithmic reduction (Log10 CFU/mL). A schematic representation is shown in Figure 1.

### 2.4 Gaseous ozone effect on *Bacillus cereus* and *Bacillus subtilis* in sessile forms

The effect of ozone gas on sessile forms was also tested, specifically how it inhibited biofilm formation or eradicated preformed biofilm. Two common food contact surfaces: polystyrene (PS) Nunc™ plates (Thermo Fisher Scientific Waltham, MA, USA), and AISI 316 stainless-steel (SS) coupons were evaluated. Biofilm was formed according to previous protocols described by Di Bonaventura et al. (2008), with slight modification (Catania et al., 2023). Briefly, bacterial strains were cultured overnight at 30°C in Brain Heart Infusion (BHI) broth (Oxoid, Basingstoke, UK), then centrifugated for 10 min at 4000 g, rinsed thrice with sterile phosphate buffer saline solution (PBS; Oxoid, Basingstoke, UK), and re-suspended in BHI broth (Oxoid, Basingstoke, UK). Cultures were diluted to reach an OD550 nm of approximately 0.125 (cell concentration of 10^8^ CFU/mL). Then, three milliliters of each diluted culture were added to 6-well Nunc™ polystyrene tissue culture plates (3 wells for each strain), with or without stainless steel coupons. Wells (in triplicate) containing only BHI broth (Oxoid, Basingstoke, UK) without bacterial inoculum, were used as negative controls.

**Figure 2:**
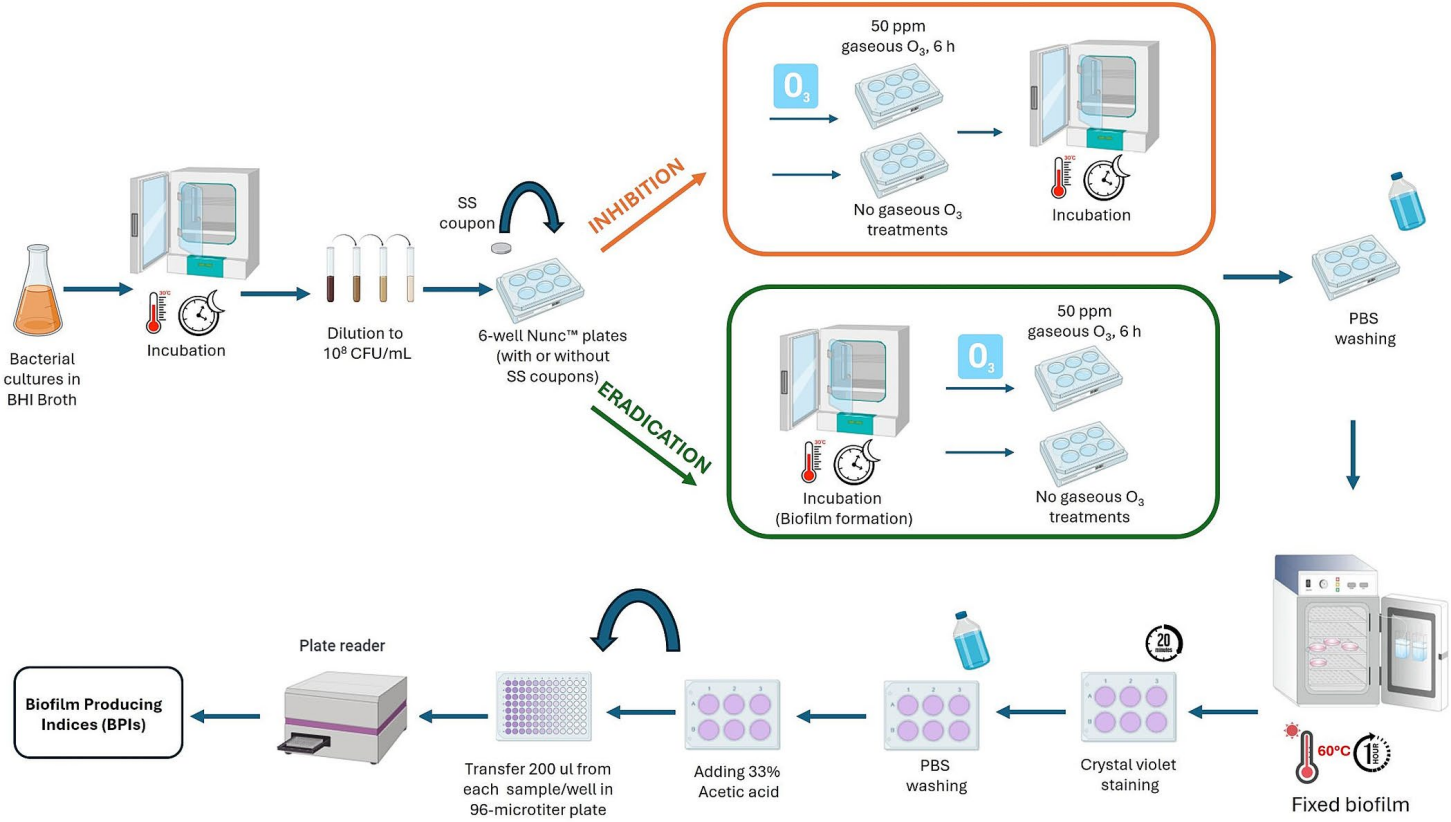
Graphic description of the experimental assay to evaluate the effect of ozone gas (50 ppm, 6 h) to inhibit and eradicate biofilm of dairy-related *B. cereus* and *B. subtilis* strains, and reference strains, at different exposure times. This image was created with BioRender (https://biorender.com/).

To evaluate the effect of ozone in inhibiting biofilm formation, 6-well Nunc™ plates with or without stainless-steel coupons were treated with gaseous ozone at 50 ppm for 6 h (Figure 2). Whereas, to assess the eradication effect, the 6-well Nunc™ plates containing *B. cereus* and *B. subtilis* cultures (cell concentration of 10^8^ CFU/mL), were firstly incubated for 24 h at 30°C to allow biofilm formation. After incubation, the cells organized in a biofilm state were treated with 50 ppm of gaseous ozone for 6 h (Figure 2). After ozone treatment, BPIs were calculated. Briefly, BHI broth (Oxoid, Basingstoke, UK) was removed from 6-well Nunc™ plates, and each well was rinsed three times with sterile PBS (Oxoid, Basingstoke, UK) to eliminate non-adherent cells. The formed biofilm was fixed at 60°C for 1 h and stained with 3 mL of a 2% crystal violet solution (95% ethanol, Sigma-Aldrich, St. Louis, MO, USA; 2% crystal violet, Sigma-Aldrich, St. Louis, MO, USA) for 20 min. After staining, wells were rinsed thrice with distilled water and dried at 37°C for 15 min. Then, 3 mL of a 33% acetic acid (Merck, Darmstadt, Germany) solution was added to each well. After 20 min, 0.2 mL from each sample were transferred to a 96-well microtiter plate (Sarstedt Srl, Milan, Italy), and the OD level of the destaining solution was measured at 490 nm in a microplate reader (iMark plate reader, Bio-Rad, Hercules, CA), see Figure 2. Results were normalized by calculating the Biofilm Production Index considering the growth area of each well:


$$BPI=\left(\frac{\mathrm{ODmean}}{\mathrm{biofilm}\;\mathrm{surface}\;\left(\mathrm{mm}2\right)}\right)\;x\;1000$$

The BPIs of treated bacteria were compared with the BPIs of controls (untreated) to evaluate the effect of ozone gas exposure in inhibiting biofilm formation.

### 2.5 SEM analysis

Reference strains *B. cereus* ATCC 14579 and *B. subtilis* NCIB 3610 were selected to visualize by scanning electron microscopy (SEM) the effect of ozone in the inhibition and the eradication (see Section 2.4) of biofilm in PS wells and SS coupons. *B. cereus* ATCC 14579 strain was selected as a low biofilm producer, as reported in this research and previous studies (Hayrapetyan et al., 2015; Kwon et al., 2017; Park et al., 2019), while *B. subtilis* NCIB 3610 was chosen for the ability to form moderate/strong biofilm, as previously described (Ostrov et al., 2019; Elegbeleye and Buys, 2020). Bacteria were grown at 30°C for 24 h on Nunc™ PS plates with or without SS coupons (as described in Section 2.4). Then, they were rinsed by dipping 3 times in sterile PBS to remove non-adherent cells and heat-fixed at 60°C for 1 h. Biomass was fixed with 2.5% glutaraldehyde (Sigma-Aldrich, St. Louis, MO, USA) in 0.1 M sodium cacodylate buffer (pH 7.2) (Sigma-Aldrich, St. Louis, MO, USA) for 30 min at room temperature and then fixed in 1% osmium tetroxide (Sigma-Aldrich, St. Louis, MO, USA) for 1 h. Samples were then rinsed with 0.1 M cacodylate buffer (Sigma-Aldrich, St. Louis, MO, USA) for 1 h to remove any unreacted glutaraldehyde (Sigma-Aldrich, St. Louis, MO, USA) before rinsing and dehydration. Samples were dehydrated with increasing concentrations of ethanol (30, 50, 70, and 80%) for 15 min for each concentration, and then three times for 15 min in 100% ethanol. Finally, they were overnight airdried. Specimens were then sputter-coated with a gold–palladium layer using an Emitech K575X Peltier-cooled (EM Technologies, Ashford, Kent, UK). Finally, selected samples were visualized using the Jeol LV300 Scanning Electron Microscope at an accelerating voltage of 25 kV and a working distance of 6 mm, with a probe current of about 100 pA. All SEM analyses were performed in two independent experiments. Samples that underwent gaseous ozone treatment were compared to control samples (no ozone treatments).

### 2.6 Statistical analysis

The data shown in this research are the average values obtained in three independent experiments with standard deviations. To assess the effect of ozone gas on bacterial cells, a two-way analysis of variance (ANOVA) with Tukey’s multiple comparison test was performed. The significant differences in BPIs before and after ozone treatments were calculated by performing a two-way ANOVA followed by a Dunnett’s multiple comparison test, while Tukey’s multiple comparison test was applied to analyze the data on culturable cells before and after the ozone exposure. Statistical analyses and graphing were performed with GraphPad Prism version 8.4.3 (GraphPad Software, San Diego, CA, USA). Differences were considered statistically significant when *p*-values were less than 0.05.

## 3 Results

### 3.1 Gaseous ozone treatment against *Bacillus cereus* and *Bacillus subtilis* cells

The results of load reduction in *B. cereus* and *B. subtilis* cells were reported in Table 2. A reduction of *B. cereus* and *B. subtilis* loads was detected after short-term exposure (10 min) and long-term exposure (1 h and 6 h) to ozone gas (50 ppm) compared to control samples.

**Table 2:** Logarithmic reduction (Log10 CFU/mL) of *B. cereus* and *B. subtilis* cells, including reference strains, compared to control samples, after 10 min, 1 h, and 6 h of ozone exposure/exposition at 50 ppm.

Strain ID	Species	Logarithmic reduction at 50 ppm		
		10 min	1 h	6 h
ATCC 14579	*B. cereus*	1.50 ± 0.20 ^a^	3.37 ± 0.10 ^b^	>7.90 ± 0.11 ^c^
BC_2		0.66 ± 0.17 ^a^	1.50 ± 0.10 ^b^	>7.30 ± 0.12 ^c^
BC_14		1.46 ± 0.15 ^a^	1.53 ± 0.10 ^a^	>7.90 ± 0.11 ^b^
BC_36		2.27 ± 0.13 ^a^	3.46 ± 0.11 ^b^	>7.50 ± 0.13 ^c^
BC_44		2.10 ± 0.20 ^a^	3.00 ± 0.10 ^b^	>7.72 ± 0.12 ^c^
NCIB 3610	*B. subtilis*	2.90 ± 0.11 ^a^	5.6 ± 0.10 ^b^	>8.11 ± 0.10 ^c^
BS_8		3.70 ± 0.10 ^a^	4.6 ± 0.10 ^b^	>8.20 ± 0.10 ^c^
BS_23		3.62 ± 0.10 ^a^	4.2 ± 0.11 ^b^	>8.30 ± 0.10 ^c^
BS_42		3.36 ± 0.16 ^a^	4.0 ± 0.11 ^b^	>8.20 ± 0.10 ^c^
BS_54		3.81 ± 0.12 ^a^	4.6 ± 0.02 ^b^	>8.30 ± 0.12 ^c^

Values represent the average ± standard deviation of three replicates. Small letters indicate differences statistically significant according to Tukey’s multiple comparison test (*p* < 0.05).

In particular, the mean logarithmic reduction ranged from 0.66 ± 0.17 and 2.27 ± 0.13 Log_10_ CFU/mL for *B. cereus* and from 2.90 ± 0.11 and 3.81 ± 0.12 Log_10_ CFU/mL for *B. subtilis* isolates after 10 min of gaseous ozone treatment. Significant differences in load reduction were observed between short (10 min) and long-term treatments (1 h and 6 h), for all strains. After 1 h of exposure to ozone gas, the logarithmic reduction was almost double for *B. cereus* strains (from 1.5 ± 0.10 to 3.46 ± 0.11), and approximately one logarithmic unit for *B. subtilis* isolates (from 4.0. ± 0.11 to 5.6 ± 0.10 Log_10_ CFU/mL), compared to 10 min-treatment. After prolonged exposure to ozone gas (6 h), no CFUs were detected (below the detection limit).

### 3.2 Gaseous ozone effect against biofilm of *Bacillus cereus* and *Bacillus subtilis* isolates in terms of inhibition and eradication

**Figure 3:**
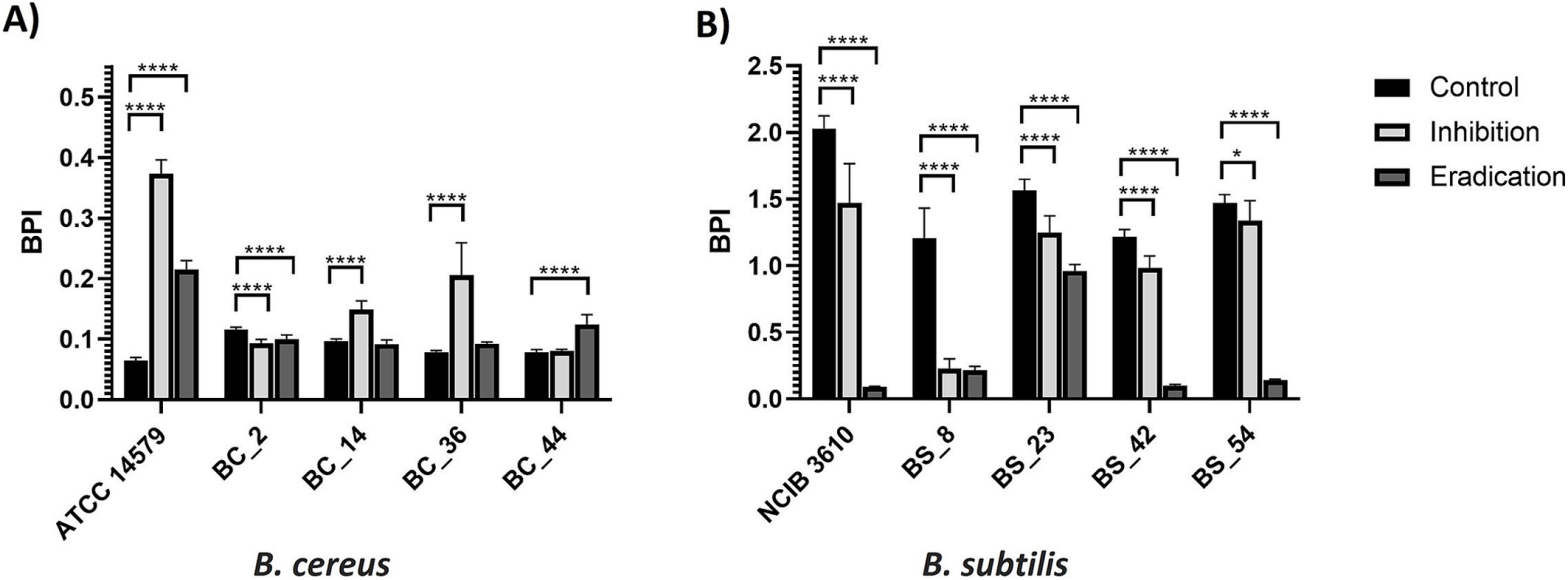
Effect of gaseous ozone at 50 ppm for 6 h on biofilm formation and eradication of **(A)** *B. cereus* and **(B)** *B. subtilis* isolates, including reference strains, in polystyrene wells. Error bars indicate standard deviation. Asterisks (*) indicate differences statistically significant according to Dunnett’s multiple comparisons. One asterisk (*) means *p* < 0.05. Four asterisks (****) indicate that the *p* < 0.0001.

**Figure 4:**
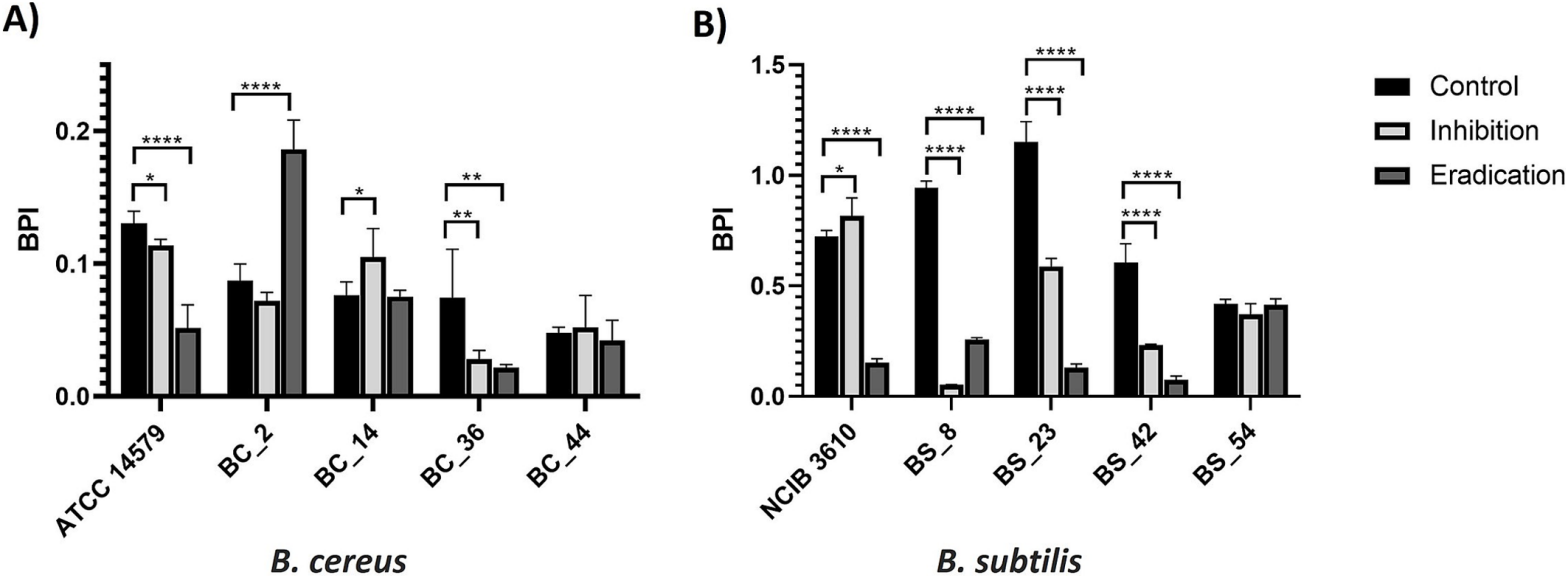
Effect of gaseous ozone at 50 ppm for 6 h on biofilm formation and eradication of **(A)** *B. cereus* and **(B)** *B. subtilis* isolates, including reference strains, in stainless steel coupons. Error bars indicate the standard deviation. Asterisks (*) indicate differences statistically significant according to Dunnett’s multiple comparisons. One asterisk (*) means *p* < 0.05. Two asterisks (**) indicate *p* < 0.01. Four asterisks (****) mean *p* < 0.0001.

The effects of gaseous ozone treatment (50 ppm) in the inhibition and eradication of biofilm of dairy *B. cereus* and *B. subtilis* isolates are summarized in Figure 3 (PS wells) and Figure 4 (SS coupons). Ozone was more effective on *B. subtilis* strains, regardless of the tested surface (Figures 3B, 4B), except in BS_54 strains for which the ozone gas treatment did not show any effect in SS (Figure 4B), and for the reference strain NCIB 3610 in which a higher BPI was observed after the inhibition ozone treatment in SS coupons (Figure 4B).

About *B. cereus* isolates, only for the BC_2 sample in PS (Figure 3A), BC_36, and the ATCC 14579 reference strain in SS (Figure 4A), the treatment with ozone gas acted on inhibition and eradication of biofilm. On the contrary, a higher BPI (compared to control) was observed after the ozone inhibition treatment (effect on biofilm formation), in ATCC 14579 and dairy BC_36 in PS (Figure 3A), and the case of dairy BC_14 both in PS (Figure 3A) than SS (Figure 4A). An increase in the BPI value of the preformed biofilm (eradication) occurred for dairy BC_44 in PS (Figure 3A) and BC_2 in SS (Figure 4A). Regarding BC_44 isolate no effect in SS coupons was observed (Figure 4A).

### 3.3 Quantification of culturable bacteria in the total biomass

Bacterial cells were counted in the biomass of each sample after gaseous ozone treatments (inhibition and eradication) in PS wells (Table 3) and SS coupons (Table 4). Significant differences in logarithmic reductions among inhibition and eradication treatments were observed for both bacterial species and surfaces (Tables 3, 4).

**Table 3:** Total CFU counts (Log CFU/mm^2^) after inhibition and eradication ozone gas treatments (50 ppm for 6 h) of *B. cereus* and *B. subtilis* isolates compared to control samples in polystyrene wells.

Strain ID	Species	Logarithmic reduction at 50 ppm in polystyrene wells	
		Inhibition	Eradication
ATCC 14579	*B. cereus*	0.00 ± 0.01 ^a^	0.50 ± 0.10 ^b^
BC_2		1.55 ± 0.07 ^b^	0.40 ± 0.08 ^a^
BC_14		0.70 ± 0.10	0.70 ± 0.10
BC_36		0.30 ± 0.15 ^a^	0.50 ± 0.15 ^b^
BC_44		0.00 ± 0.15	0.00 ± 0.11
NCIB 3610	*B. subtilis*	0.84 ± 0.09 ^a^	2.77 ± 0.14 ^b^
BS_8		1.95 ± 0.12	1.97 ± 0.15
BS_23		0.62 ± 0.16 ^b^	0.23 ± 0.10 ^a^
BS_42		0.00 ± 0.05 ^a^	2.70 ± 0.10 ^b^
BS_54		0.10 ± 0.10 ^a^	1.90 ± 0.10 ^b^

Each value was the result of the average of three biological experiments and the standard deviation for each strain. To compare the number of sessile cells yielded among strains, ANOVA and Tukey’s *post hoc* test were performed. Groups with different alphabets indicate significant differences (*p* < 0.05).

**Table 4:** Total CFU counts (Log CFU/mm^2^) after inhibition and eradication ozone treatments (50 ppm for 6 h) of *B. cereus* and *B. subtilis* isolates compared to control samples in stainless steel coupons.

Strain ID	Species	Logarithmic reduction at 50 ppm in stainless steel coupons	
		Inhibition	Eradication
ATCC 14579	*B. cereus*	3.10 ± 0.10 ^b^	1.30 ± 0.10 ^a^
BC_2		1.70 ± 0.20 ^b^	0.10 ± 0.09 ^a^
BC_14		0.12 ± 0.11 ^a^	0.43 ± 0.10 ^b^
BC_36		0.20 ± 0.10 ^b^	0.00 ± 0.10 ^a^
BC_44		0.30 ± 0.21	0.30 ± 0.20
NCIB 3610	*B. subtilis*	0.69 ± 0.21 ^a^	2.70 ± 0.12 ^b^
BS_8		2.12 ± 0.15 ^b^	0.27 ± 0.09 ^a^
BS_23		0.35 ± 0.24 ^a^	2.00 ± 0.13 ^b^
BS_42		0.38 ± 0.11 ^a^	1.06 ± 0.10 ^b^
BS_54		0.27 ± 0.24 ^b^	0.00 ± 0.20 ^a^

Each value was the result of the average of three biological experiments and the standard deviation for each strain. To compare the number of sessile cells yielded among strains, ANOVA and Tukey’s *post hoc* test were performed. Groups with different alphabets indicate significant differences (*p* < 0.05).

For *B. cereus* strains, ozone gas acts better in inhibiting biofilm formation than in eradicating it, with a stronger action on SS. The mean reduction of *B. cereus* viable cells after inhibition treatment (see Section 2.4) was 0.51 ± 0.10 Log CFU/mm^2^ in PS wells (Table 3) and 1.08 ± 0.14 Log CFU/mm^2^ in SS coupons (Table 4). Whereas, after the eradication treatments (see Section 2.4), an average reduction of 0.42 ± 0.11 Log CFU/mm^2^ was detected both in PS wells (Table 3) and SS coupons (Table 4).

Concerning *B. subtilis* isolates, ozone gas had the most pronounced effect in the eradication of biofilm, especially on PS. After the inhibition treatment (see Section 2.4), similar values in the reduction average were reported in PS wells (0.70 ± 0.10 Log CFU/mm^2^, see Table 3) and SS coupons (0.76 ± 0.19 Log CFU/mm^2^, see Table 4). Whereas, a greater reduction was observed after eradication treatment (see Section 2.4), around a mean of 1.91 ± 0.12 Log CFU/mm^2^ in PS wells (Table 3) and 1.21 ± 0.13 Log CFU/mm^2^ in SS coupons (Table 4).

### 3.4 SEM

**Figure 5:**
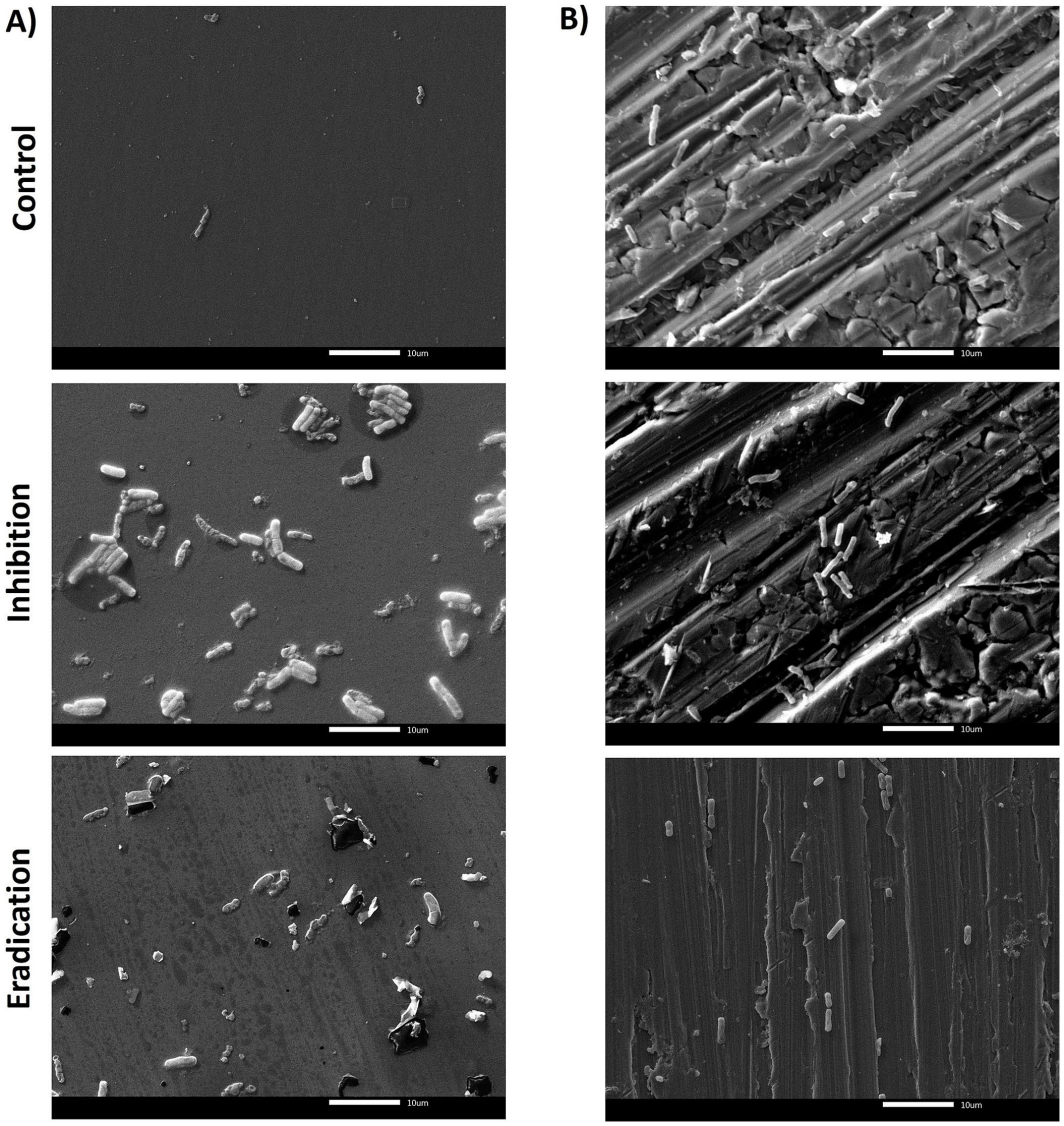
SEM micrographs of biofilms formed by *B. cereus* ATCC 14579 reference strain in **(A)** polystyrene and **(B)** stainless steel, after 6 h of exposure to 50 ppm of gaseous ozone. The control group (top of the figure) represents the untreated biofilm. The central/middle images represented the effect of ozone on inhibiting ATCC 14579 biofilm formation. The downward figures show the effect of gaseous ozone on preformed biofilm (eradication). Magnification 2000x.

Scanning electron microscopy (SEM) analysis was performed to visualize the effect of ozone treatment (50 ppm for 6 h) in terms of inhibition of biofilm formation and eradication of pre-formed biofilm on both surfaces (PS wells and SS coupons). The reference strains *B. cereus* ATCC 14579 and *B. subtilis* NCIB 3610 were selected as representative isolates for this analysis. The *B. cereus* ATCC 14579 strain, both on polystyrene and stainless steel, showed the presence of bacterial cells but no polymeric matrix (Figure 5). This result agrees with the low BPI values found on both surfaces (see Section 3.2).

**Figure 6:**
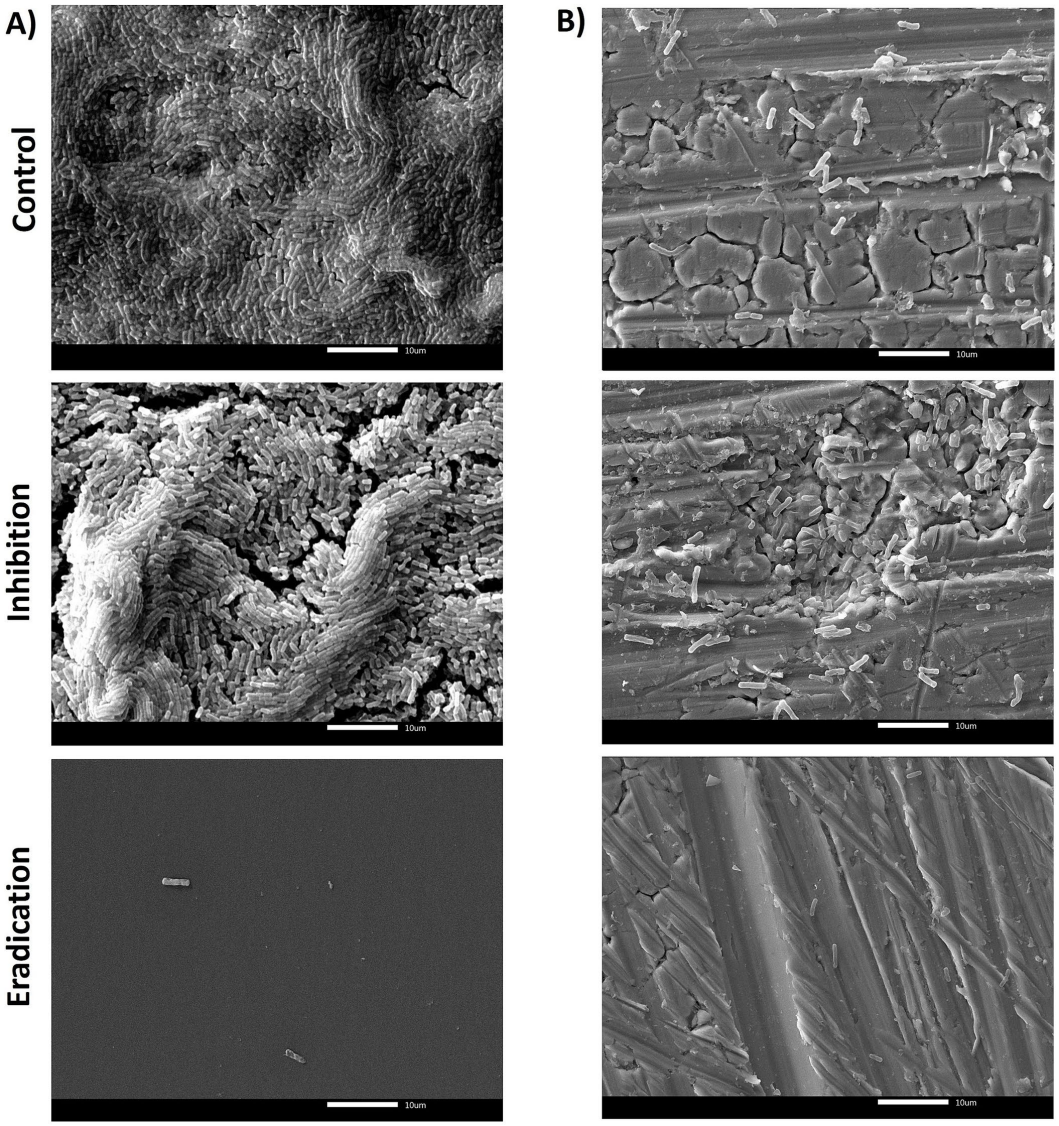
SEM micrographs of biofilms formed by *B. subtilis* NCIB 3610 reference strain in **(A)** polystyrene and **(B)** stainless steel, after 6 h of exposure to 50 ppm of gaseous ozone. The control group (top of the figure) represents the untreated biofilm. The central/middle images represented the effect of ozone on inhibiting NCIB 3610 biofilm formation. The downward figures show the effect of gaseous ozone on preformed biofilm (eradication). Magnification 2000x.

In PS, SEM observations revealed that the untreated biofilms of *B. subtilis* NCIB 3610 reference strain were dense, with a well-organized structure, and a greater number of adherent cells on the surface than *B. cereus* isolates (Figure 6A). As shown in Figure 4A, eradication was the most effective treatment, leaving only a few microorganisms after ozone exposure, which aligns with the BPI data (see Section 3.2). The ability to form biofilm on SS was lower compared to PS (Figure 6B), in agreement with BPI values, but even in this case, it seemed that ozone acted better in eradicating the biofilm rather than in inhibiting it (Figure 6B).

## 4 Discussion

The monitoring and prevention of bacterial contamination is a major challenge for the dairy industry. Gaseous ozone is a promising solution in the dairy industry to control bacterial biofilm of spoilage and pathogenic bacteria in equipment and machinery employed in dairy plants such as homogenizers, separators, pasteurizers, and milk tanks (Panebianco et al., 2022). The effectiveness of ozone against microorganisms depends on several factors, including their type (Gram-positive or Gram-negative), state (free-living or sessile), and the nature of the surface (Sharma and Hudson, 2008; Martinelli et al., 2017). To date, a few studies investigated the effect of ozone gas in preventing and/or removing bacterial biofilm.

In this research, we evaluated the effect of gaseous ozone (50 ppm) on dairy *B. cereus* and *B. subtilis* cells at short (10 min) and long (1 h and 6 h) times of exposure. We observed that the antimicrobial effect increased significantly with exposure time. Even after 10 min of ozone exposure, a reduction in total bacterial load was observed, with an average of around 1.60 ± 0.17 Log_10_ CFU/mL for *B. cereus* and more pronounced for *B. subtilis* isolates around 3.48 ± 0.12 Log_10_ CFU/mL. After 1 h of treatment, there was a significant (*p* < 0.05) increase of one logarithmic unit for both bacterial species, with a reduction of roughly 2.57 ± 0.10 Log_10_ CFU/mL and 4.60 ± 0.09 Log_10_ CFU/mL, respectively for *B. cereus* and *B. subtilis* isolates. Similar results were reported in a study conducted on *L. monocytogenes* cells, in which after 10 min and 30 min of treatment with ozone gas, the antimicrobial effect was around 3.70 ± 0.40 and 3.90 ± 0.40 Log_10_ CFU/mL, respectively (Panebianco et al., 2021). Sharma and Hudson (2008) evaluated the effect of a lower ozone concentration (25 ppm) on planktonic cells of Gram-negative and Gram-positive microorganisms, including *B. cereus* ATCC 11778 strains (Sharma and Hudson, 2008). After 20 min-exposure, a reduction >4 Log_10_ CFU/ml was detected for all Gram-negative species tested, while lower values (about 3 Log_10_ CFU/ml) were assessed for Gram-positive bacteria, including *B. cereus* ATCC 11778 (Sharma and Hudson, 2008). In this study, prolonged treatment up to 6 h resulted in complete inactivation of cells, in both bacterial species (no CFUs were detected).

It has been shown that high concentrations of ozone are required to have an effect against bacterial biofilms. A study by Harada and Nascimento (2021a) demonstrated the effectiveness of treatment with gaseous ozone at 45 ± 2 ppm against *B. cereus* biofilms on stainless steel and polypropylene (PP) (Harada and Nascimento, 2021a). In another research, ozone gas at 45 ± 2 ppm reduced *L. monocytogenes* biofilm cell counts formed on PP after 5 min of treatment (Harada and Nascimento, 2021b). In contrast, Panebianco et al. (2021) observed a partial effect on *L. monocytogenes* biofilm using high ozone concentration (50 ppm) and prolonged exposure (6 h). Considering the literature data, and the higher resistance to oxidative stress of bacterial cells organized in a biofilm state, compared to free-living bacterial cells (Bialka and Demirci, 2007; Marino et al., 2018), a high ozone concentration (50 ppm) and long exposure time (6 h) were applied against the biofilm produced by dairy Bacillus isolates on PS and SS. Polystyrene is a hydrophobic substrate extensively used for packaging food products (Pilevar et al., 2019), whereas stainless steel is a hydrophilic substrate widely employed in food processing facilities and equipment, such as tanks and pipes (Dewangan et al., 2015). The 6-well PS plates (with and without SS coupons) were used for biofilm development. This system overcomes the limitation of the most used approaches (microtiter plate assay-96 wells format) for evaluating the biofilm-forming ability of bacterial isolates, concerning possible nutrient limitation and total surface area exposed for biofilm formation. The biofilm production indices of *B. cereus* and *B. subtilis* isolates were expressed as BPI values and agreed with the results obtained in previous work (Catania et al., 2023). *B. subtilis* isolates showed higher BPIs on both surfaces, compared to *B. cereus* strains (Figures 4, 5).

For each bacterial isolate, the effect of ozone gas was tested to assess its ability to inhibit biofilm formation and eradicate an established biofilm on the two surfaces (see Section 2.4). Variability was observed between the two different species organized in biofilm.

*B. subtilis* strains were generally more sensitive than *B. cereus* isolates to gaseous ozone treatment at 50 ppm for 6 h (see Figures 3B, 4B), except for the dairy isolate BS_54, where ozone gas had no effect on the biofilm formed on SS coupons (see Figure 4B). In previous research, ozone was applied at a lower concentration (1.4 ppm), in combination with an alkaline cleaning-in-place reagent (NaOH), against biofilms formed by *B. subtilis* and *B. amyloliquefaciens* isolates on stainless steel coupons (Tiwari et al., 2017). Results indicate increased inactivation of biofilms within 1 min for *B. amyloliquefaciens* and 2 min for *B. subtilis* strain using a combination of 1.4 ppm of ozone and 1% NaOH, while application of 1% NaOH alone took 4 min to completely remove the biofilm from SS coupons (Tiwari et al., 2017).

A greater variability was observed for *B. cereus* isolates, in which ozone was effective on dairy BC_2 isolate in PS (Figure 3A), on ATCC 14579 reference strain, and on dairy BC_36 isolate in SS coupons (Figure 4A). In a recent study, Harada and Nascimento (2021a) tested dry sanitizing methods, including gaseous ozone, at 45 ppm on *B. cereus* biofilm on polypropylene and stainless steel. After 30 min of exposure, a slight reduction of biofilm was observed, with a better effect on SS than on PP (Harada and Nascimento, 2021a). A significant increase of BPI was observed in the inhibition phase for dairy BC_14 both in PS (Figure 3A) and SS (Figure 4A), and for BC_36 isolate in PS (Figure 3A). For the dairy strains BC_2 and BC_44, a higher BPI, compared to control, was observed on preformed biofilm (eradication), respectively in SS (Figure 4A) and PS (Figure 3A). The ATCC 14579 reference strain was found to be sensitive to the effect of ozone on SS (Figure 4A), while on PS an increase in BPI, after ozone treatment, was assessed in both the inhibitory and pre-formed biofilm (Figure 3A). The reason for the different sensitivity to ozone by the two bacterial species is unclear and further investigations are certainly needed to explain this behavior. It is known that the biofilm matrix produced by microorganisms consists of several components: extracellular DNA (eDNA), proteins, lipids, and exopolysaccharides (Flemming et al., 2016). The extra polymeric substance (EPS) is one of the main components of biofilm and plays a key role in promoting microbial adhesion to surfaces. In some cases, it can account for up to 90% of the biofilm biomass (Nguyen et al., 2020). It constitutes the biofilm’s scaffolding and contributes to its function (Flemming and Wingender, 2010). The structure and composition of biofilm and EPS can vary considerably depending on microorganisms (Flemming et al., 2016). The biofilm structure of *B. cereus* isolates is not completely known (Majed et al., 2016), and distinct patterns concerning composition, structure, and physicochemical properties were highlighted among *B. cereus* strains (Lim et al., 2021). *B. cereus* biofilm, when formed at the air-liquid interface, comprises a ring strongly adhered to the wall and a pellicle showing protrusions (Majed et al., 2016). On the contrary, the biofilm of *B. subtilis* isolates has a very characteristic appearance, typically formed by many wrinkles (Cairns et al., 2014). Biofilm formation in *B. subtilis* isolates is regulated by the *epsA-O* operon that encodes for the exopolysaccharides’ production (Kearns et al., 2005). The *epsA-O* deletion leads to a poorly structured and fragile biofilm (Lemon et al., 2008). The *eps* locus is also present in the *B. cereus* group, but unlike *B. subtilis*, the deletion of *eps* does not affect the biofilm formation (Gao et al., 2015). These significant differences between the two bacterial species, in terms of biofilm structure, and genetic determinants regulating EPS, are also reflected in the different behavior toward the action of ozone gas. The increase in biomass, observed in this study in most *B. cereus* isolates after ozone gas treatment, could represent a form of resistance to ozone-induced oxidative stress, which would lead microorganisms to increase the production of extra polymeric substance. The biofilm matrix is known for its formidable barrier effect on exogenous stressors (Nadell et al., 2015). This phenomenon was previously observed on dairy *L. monocytogenes* isolates, after 6 h of ozone gas exposure at 50 ppm on PS (Panebianco et al., 2021). Previous studies highlighted that EPS may be secreted in severe conditions (Jefferson, 2004; Cai et al., 2021). It has been seen that some antimicrobial compounds at sub-lethal concentrations can promote EPS formation (Kaur and Dey, 2023). In recent research carried out on *P. aeruginosa* strains, the authors underlined that the oxidative stress response of bacteria in the sessile state to the sublethal stress of photocatalysis (PC), was much stronger than in control groups, observing the promotion of secretion of EPS (Chen et al., 2022). Bacterial ATP levels were also measured since in bacteria ATP is mainly secreted by polysaccharides and proteins, which are the main constituents of EPS (Flemming and Wingender, 2010). An increase in ATP production was observed, suggesting that exposure to harsh conditions not only enhances EPS secretion but also stimulates bacterial activity (Chen et al., 2022). EPS may represent a form of biofilm resilience to antimicrobial agents. The incomplete removal of biofilm in industrial environments, and thus its persistence, may enhance resistance to further disinfection through increased production of exopolysaccharides (Bridier et al., 2011). In general, the bacterial resistance to antimicrobials mediated by EPS, may increase oxidative stress response, and enhance mutation in genes coding for biofilm. Further investigations should be carried out to elucidate the increase in BPI values (biomass) in *B. cereus* isolates after ozone gas treatment, including the expressions of oxidative stress and biofilm-related genes.

The quantification of biofilm biomass was consistent with SEM observations. In general, the action of ozone gas was weaker for *B. cereus* isolates, as for the reference strain ATCC 14579 (Figure 5). In contrast, there was a more marked effect in *B. subtilis* isolates, including the NCIB 3610 reference strain, both in preventing biofilm formation (inhibition) and against preformed biofilm (eradication) (Figure 6).

The counts of adherent bacteria in established biofilms were performed to evaluate a correlation between the biomass and the number of culturable bacteria after ozone treatment. Results showed a reduction of microbial loads after the ozone treatment, compared to control (untreated) samples. The cell reduction was not correlated with decreases in BPIs. A not-direct correlation between biofilm biomass and viable bacterial cells was highlighted in other studies (Monteiro et al., 2015; Stiefel et al., 2016; Rodrıiguez-Lázaro et al., 2018). A possible explanation could be related to the method applied to measure the total biomass, since crystal violet staining includes both viable and non-viable bacteria, as well as the polymeric matrix cells (Monteiro et al., 2015; Stiefel et al., 2016; Rodrıiguez-Lázaro et al., 2018), while CFU excludes dead bacteria and debris, and only viable bacteria are counted.

Our research focused on dairy *B. cereus* and *B. subtilis* isolates, whose presence is a factor of great concern in the food industry, since they are pathogenic (*B. cereus*) and biofilm-forming microorganisms, that can be easily dispersed in the manufacturing environment and contaminated food products. We observed that gaseous ozone was effective against *B. cereus* and *B. subtilis* cells. On the other hand, its action against consolidated biofilm was variable. Gaseous ozone was effective in inhibiting and eradicating *B. subtilis* biofilm, but it was not effective against *B. cereus* biofilm. Taking into account the results obtained in this study, we considered the application of ozone gas as a complementary step to cleaning-in-place operations to enhance its efficacy. Specifically, its practical employment should be envisaged after CIP since ozone gas is toxic at high concentrations. For this reason, its use should be scheduled at the end of the working day, in the absence of food handlers.

## 5 Conclusion

Ozone has been studied for many years for its biocidal activity. The main benefit of gaseous ozone use in the food sector is related to the absence of residues due to its accelerated decay. However, a limit for its use is represented by the toxicity at high concentrations for human health. The advantage of using gaseous ozone, compared to traditional methods, is related to its ability to easily spread, and therefore reach even the niches, typical of an industrial plant, which are difficult to reach for normal disinfection procedures, and where biofilm-forming bacteria can settle.

Although additional studies are needed to support the potential application of this promising technology in dairy processing plants, our data indicate that the gaseous ozone treatment (50 ppm) has an antibacterial effect on dairy *B. cereus* and *B. subtilis* cells at different times of exposure (10 min, 1 h, and 6 h). Conversely, the effect of ozone gas (50 ppm) on a long exposure time (6 h) was effective against the established biofilm of *B. subtilis* isolates, whereas a partial or no effect was observed against the biofilm of *B. cereus*. Considering the results, ozone gas could be used not alone, but in association with the CIP system to improve the cleaning/disinfection routine procedures against *Bacillus* spp. isolates.

Lastly, it might be interesting to evaluate the performance of gaseous ozone in different experimental conditions (for example on biofilm formation under dynamic conditions) to simulate the environment typically encountered in dairy processing plants and to analyze its effectiveness against spores.

## Data Availability

The raw data supporting the conclusions of this article will be made available by the authors, without undue reservation.
